# Perioperative mental health needs in urological and genitourinary surgery: a scoping review

**DOI:** 10.3389/fpubh.2026.1904213

**Published:** 2026-08-13

**Authors:** Qiwei Wang, Dingyang Lv, Huiyu Zhou, Zhengkun Wang, Jinshuai Li, Zhiwei Guo, Weibing Shuang

**Affiliations:** 1 First Clinical Medical College, Shanxi Medical University, Taiyuan, China; 2 Department of Urology, The First Hospital of Shanxi Medical University, Taiyuan, China

**Keywords:** genitourinary surgery, mental health services, perioperative mental health, psychological distress, public mental health, scoping review, supportive care, urological surgery

## Abstract

**Introduction:**

Perioperative psychological distress may affect surgical recovery, yet evidence specific to urological and genitourinary procedures remains fragmented. These procedures can affect urinary control, sexual function, genital integrity, body image, and long-term self-management. This scoping review mapped reported mental health problems, associated risk factors and supportive interventions, and examined their implications for perioperative care pathways.

**Methods:**

This scoping review was reported in accordance with the Preferred Reporting Items for Systematic Reviews and Meta-Analyses extension for Scoping Reviews. PubMed, Embase, Web of Science, ScienceDirect, PsycINFO, and CINAHL were searched for English-language studies published from January 2000 to May 2026. Two reviewers independently screened the studies and charted the data, and the findings were synthesized descriptively using evidence mapping.

**Results:**

Forty-four studies were included. Most evidence came from adult oncological populations, and observational designs predominated. The heterogeneous evidence could be organized into a provisional staged pattern comprising preoperative concerns related to uncertainty and fear, early postoperative concerns related to pain, sleep, and device adaptation, and longer-term concerns related to urinary, sexual, body-image, and social adjustment. Longer-term distress was concentrated in selected subgroups and was commonly linked to urinary diversion, incontinence, sexual dysfunction, altered genital anatomy, body-image concerns, social constraints, limited support, self-perceived burden, and prior mental health vulnerability. Reported supportive approaches included prehabilitation, structured education, multidisciplinary or pathway-based care, digital and virtual reality strategies, transcutaneous electrical acupoint stimulation, multimodal analgesia, and opioid stewardship.

**Discussion:**

The available evidence remains heterogeneous and largely observational. These findings identify candidate components for the future development and prospective evaluation of perioperative mental health pathways, including repeated assessment, structured education, family-inclusive support, and referral mechanisms.

## 1 Introduction

Surgery is a potent psychosocial stressor, and psychological distress during the perioperative period is increasingly recognizedas a clinically relevant aspect of recovery rather than a secondary emotional reaction. Patients may experience uncertainty about treatment outcomes, fear of pain and anesthesia, sleep disturbance, depressive symptoms, and concerns about postoperative functioning and social reintegration (1–3). Accumulating evidence suggests that perioperative anxiety and depression are common and potentially modifiable, and are associated with greater symptom burden, poorer functional recovery, and worse health-related quality of life in surgical and oncological settings (4–6). Perioperative psychological distress is therefore a care-relevant factor that may influence communication, symptom appraisal, treatment engagement, self-management, and recovery across the surgical pathway.

Perioperative mental health may be particularly important in urological and genitourinary surgery because the diseases and procedures involved often affect urinary control, sexual function, genital integrity, reproductive identity, and body image. In addition to the psychological impact of a cancer diagnosis, postoperative changes such as urinary diversion, incontinence, erectile or sexual dysfunction, altered genital or urinary anatomy, and dependence on devices such as stomas, catheters, or ureteral stents may challenge self-concept, intimate relationships, family roles, and social participation (7–10). These concerns are relevant across the life course and across different urological populations, including children and adolescents with urological conditions (11, 12), adults and older patients undergoing benign or oncological procedures (7–11), and transgender and gender-diverse individuals undergoing gender-affirming surgery (13–17). Many of these concerns are encountered in routine care settings where perioperative teams must provide education, symptom monitoring, continence and stoma support, discharge preparation, referral coordination, and follow-up.

Despite growing recognition of mental health needs in surgical and urological populations, the evidence remains fragmented across procedure types, perioperative time points, psychological measures, and supportive interventions (18, 19). This fragmentation limits the extent to which existing evidence can inform practical questions of care delivery, including when screening should occur, which patients should be prioritized for intensified support, what concerns should trigger referral, and how psychosocial support can be integrated into perioperative, rehabilitation, and follow-up pathways (20, 21). This scoping review aimed to map perioperative mental health problems, associated risk factors and supportive interventions in urological and genitourinary surgery, and to identify implications for screening, referral and supportive care across the surgical pathway.

## 2 Methods

### 2.1 Design

This scoping review was conducted to map the breadth, characteristics and clinical relevance of evidence on perioperative mental health in urological and genitourinary surgery. The review question was: What perioperative mental health problems, risk factors and supportive interventions have been reported among people undergoing urological or genitourinary surgery or invasive urological procedures? The review was guided by Joanna Briggs Institute methodology for scoping reviews and reported in accordance with the Preferred Reporting Items for Systematic Reviews and Meta-Analyses extension for Scoping Reviews. An *a priori* protocol was developed before study screening. Because the review was initially undertaken as an internal evidence-mapping project, prospective registration was not planned at inception. The absence of prospective registration is acknowledged as a limitation.

Given the substantial heterogeneity in study designs, surgical procedures, perioperative time points, psychological outcome measures and reporting formats, quantitative pooling was not undertaken. Instead, the review used evidence mapping and descriptive synthesis to summarize the available literature and identify implications for perioperative screening, risk stratification, referral and supportive care pathways.

### 2.2 Eligibility criteria

#### 2.2.1 Population

Studies were eligible if they included patients undergoing urological and genitourinary surgery or invasive urological procedures. Eligible populations included, but were not limited to, patients undergoing oncological urological and genitourinary surgery, urinary diversion, prostate surgery, renal surgery, endoscopic or device-related urological procedures, living kidney donation, kidney transplantation, benign reconstructive urology, pediatric urology or gender-affirming surgery involving urological or genital procedures.

#### 2.2.2 Concept

Studies were required to report at least one mental health-related outcome. Eligible outcomes included anxiety, depression, psychological distress, perceived stress, mental health service use, suicide-related outcomes, emotional or mental domains of health-related quality of life, body image-related distress, sexual function-related distress, gender-role or masculinity-related concerns, self-perceived burden, sleep disturbance, fatigue, coping resources or social support when these were analyzed in relation to perioperative psychological health.

#### 2.2.3 Context

Studies were eligible if outcomes were assessed at one or more perioperative time points, including the preoperative phase, intraoperative or procedural period, early postoperative recovery, discharge or rehabilitation period, or medium- to longer-term postoperative follow-up.

Original research articles using quantitative, qualitative or mixed-methods designs were eligible. Review articles, systematic reviews, narrative reviews, case reports, corrigenda, editorials, commentaries, non-original articles, studies unrelated to urological and genitourinary surgery, studies without a perioperative time point and studies without mental health-related outcomes were excluded.

### 2.3 Information sources and search strategy

A systematic literature search was performed in PubMed, Embase, Web of Science, ScienceDirect, PsycINFO and CINAHL. The search was limited to English-language publications published from January 2000 to May 2026. These databases and search platforms were selected to provide complementary coverage of biomedical and surgical research, psychological literature, and nursing and allied-health research. The final search was conducted in May 2026. The search strategy was developed to capture literature on perioperative mental health outcomes in patients undergoing urological and genitourinary surgery or invasive urological procedures. Search terms combined concepts related to perioperative timing and mental health-related outcomes. Urological and genitourinary relevance was determined during screening according to the predefined eligibility criteria. Full search terms and database-specific combinations are provided in Supplementary Appendix S1.

### 2.4 Study selection

All records identified through database searching were imported into reference management software, and duplicates were removed before screening. Titles and abstracts were screened independently by two reviewers against the predefined eligibility criteria. Full texts of potentially eligible articles were then retrieved and assessed independently by the same two reviewers. Disagreements at either stage were resolved through discussion and consensus; when consensus could not be reached, a third reviewer was consulted.

**Figure 1:**
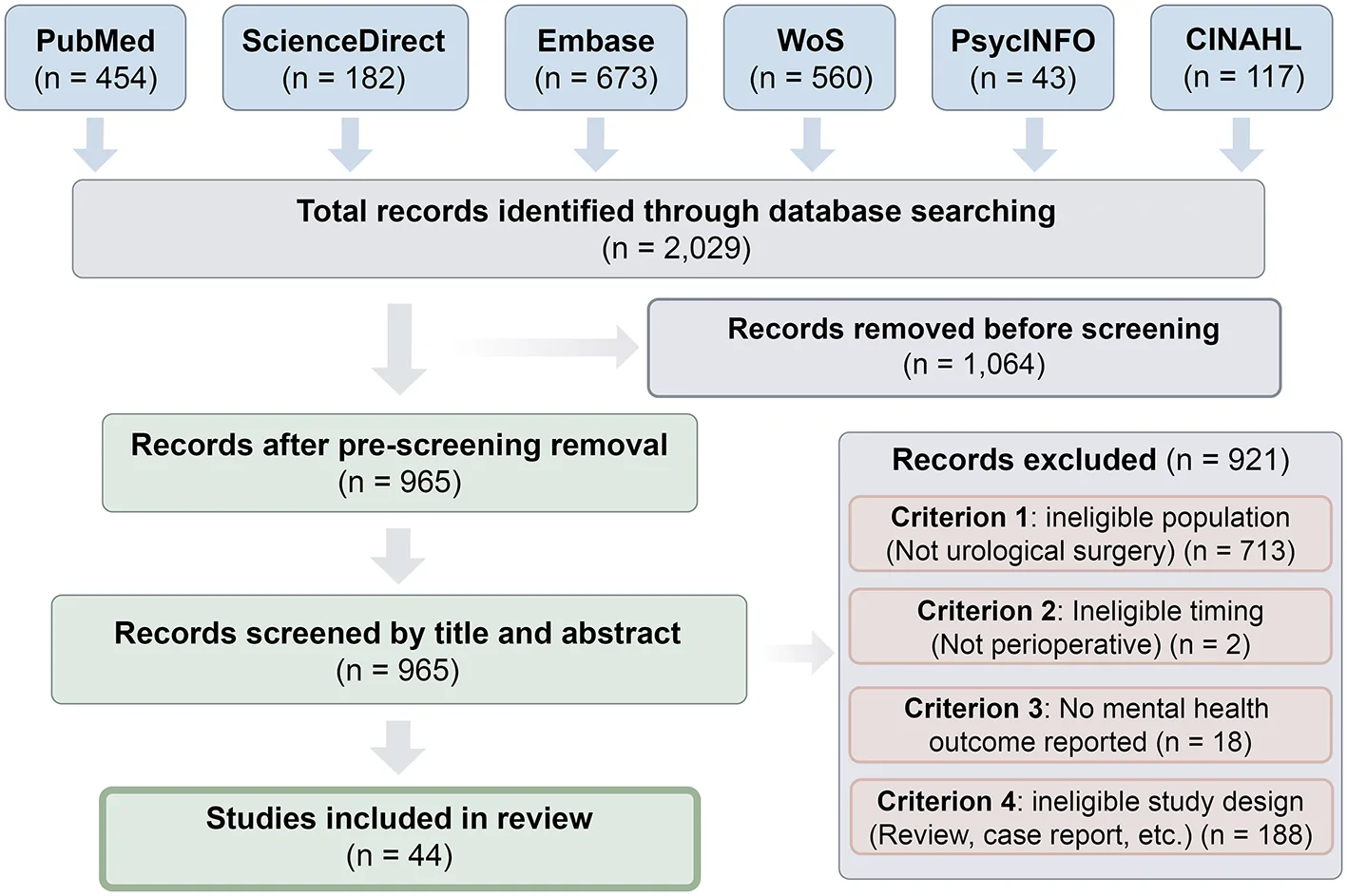
Flow diagram of the literature search and study selection process.

Database searching identified 2,029 records, including 454 from PubMed, 182 from ScienceDirect, 673 from Embase, 560 from Web of Science, 43 from PsycINFO and 117 from CINAHL. Before screening, 1,064 records were removed, comprising 1,059 duplicate records, one record published outside the predefined date range and four non-English records. A total of 965 records entered the eligibility process consisting of title and abstract screening followed by full-text assessment of potentially relevant reports. Across these stages, 921 records or reports were excluded: 713 because the population was ineligible or not related to urological or genitourinary surgery, two because the timing was not perioperative, 18 because no eligible mental health-related outcome was reported, and 188 because of ineligible study design, including reviews, case reports or other non-eligible article types. Finally, 44 full-text studies met the inclusion criteria and were included in the review. The study selection process is presented in Figure 1.

### 2.5 Data charting

Data were charted using a standardized extraction form developed for this review. The charting form was piloted on a subset of included studies and refined before full data extraction. Extracted items included author and year, country or region, study design, population characteristics, procedure type, sample size, perioperative time point, psychological instruments or data sources, mental health-related outcomes, comparator or control condition where applicable, follow-up duration, attrition information, key findings, clinical implications and major methodological limitations.

Data charting was performed independently by two reviewers. Discrepancies were resolved by discussion and consensus. When necessary, a third reviewer was consulted. Relevant study characteristics, methodological features and key findings are summarized in Supplementary Table S1. Supplementary Table S1 also identifies the synthesis domain or domains to which each study contributed, allowing the narrative findings to be traced to the underlying studies. A detailed inventory of psychological measures used across the included studies is provided in Supplementary Table S2.

### 2.6 Synthesis

Findings were synthesized descriptively using evidence mapping and narrative synthesis. The synthesis was organized around five domains: study selection and characteristics; mental health outcomes and assessment approaches; the temporal trajectory of perioperative psychological distress; urology-relevant risk domains; and supportive interventions with implications for care pathway design. This structure was used to identify evidence patterns and gaps relevant to public mental health, perioperative screening, risk stratification, supportive education, referral, service delivery and longitudinal follow-up.

### 2.7 Critical appraisal and methodological interpretation

Consistent with the purpose of a scoping review, formal risk-of-bias assessment was not used as a criterion for study exclusion. However, methodological features relevant to interpretation were charted and considered during synthesis. These included study design, sample size, procedure type, population characteristics, timing of assessment, psychological instruments used, comparator availability where applicable, follow-up duration, attrition information and major methodological limitations.

These features were used to interpret the strength, consistency and applicability of the evidence, particularly when distinguishing descriptive or associative findings from intervention-related evidence. Methodological features of the included studies are summarized in Supplementary Table S1.

## 3 Results

### 3.1 Study selection and study characteristics

A total of 44 studies met the inclusion criteria and were included in the evidence map. The included evidence covered a broad range of urological conditions and perioperative settings, including oncological surgery for bladder, prostate, renal and penile cancer; radical cystectomy and urinary diversion; diagnostic or device-related urological procedures; living kidney donation; kidney transplantation; benign reconstructive urology; pediatric urology; and gender-affirming surgery. Most studies were conducted in adult oncological populations, particularly patients undergoing treatment for bladder cancer, prostate cancer or other genitourinary malignancies. Fewer studies focused on benign procedures, transplantation-related care, pediatric urology or gender-affirming surgery.

The evidence base was methodologically heterogeneous. Observational designs predominated, including cross-sectional surveys, retrospective cohorts, prospective cohorts, registry-based analyses, longitudinal patient-reported outcome studies and qualitative studies. Controlled or interventional studies were less common and included small randomized or feasibility trials, perioperative nursing interventions, prehabilitation programs, digital interventions and non-pharmacological perioperative strategies. Repeated-measure and longitudinal designs provided important information on recovery trajectories after radical cystectomy, radical prostatectomy, nephrectomy and rehabilitation, but many studies still assessed mental health at a single time point.

Of the 44 included studies, 24 (54.5%) focused primarily on adult oncological populations, particularly bladder and prostate cancer. Thirty-six studies (81.8%) were observational or qualitative, whereas eight (18.2%) evaluated an intervention or structured care approach; 27 studies (61.4%) were explicitly identified as single-center. Evidence concerning pediatric urology, benign and reconstructive conditions, transplantation, living kidney donation, urogynecology and gender-affirming surgery was comparatively limited. Overall, the available evidence was broad in clinical scope but uneven in depth, with stronger descriptive and associative evidence than comparative effectiveness evidence.

### 3.2 Mental health outcomes and assessment approaches

Anxiety and depression were the most frequently assessed mental health outcomes. The Hospital Anxiety and Depression Scale was widely used in studies of urological cancer, living kidney donation, benign prostatic hyperplasia, kidney transplantation and perioperative digital interventions (7, 22–29). Other commonly used instruments included the Zung Self-Rating Anxiety Scale and Self-Rating Depression Scale in studies of prostate cancer, kidney transplantation and newly diagnosed bladder or kidney cancer (30–33); the State-Trait Anxiety Inventory in studies of urological cancer surgery and procedures under local anesthesia (7, 34); the Generalized Anxiety Disorder 7-item scale and Patient Health Questionnaire instruments in studies of urological cancer and gender-affirming surgery (16, 35, 36); and the Center for Epidemiologic Studies Depression Scale or Beck Depression Inventory in studies of living kidney donation and newly diagnosed urological malignancy (30, 37). The Amsterdam Pre-Operative Anxiety and Information Scale (APAIS), including a study-modified postoperative version, was used to assess anxiety and information needs in patients undergoing major urogynecologic surgery (38). Visual analog scales were also used for short-term perioperative anxiety, distress or procedure-related discomfort (24, 34, 39).

Beyond anxiety and depression, several studies assessed broader psychological and functional domains. Health-related quality of life was commonly measured using generic instruments such as the SF-36 or related mental health domains, especially in studies of living kidney donors, benign ureteral reconstruction, benign prostatic hyperplasia, pelvic exenteration, cystectomy, prostatectomy and adrenalectomy (28, 29, 37, 40–43). Cancer-specific distress, perceived stress and acute distress were assessed using instruments such as the Questionnaire on Stress in Cancer Patients, Distress Thermometer and Perceived Stress Scale (40, 44, 45). Other outcomes included self-esteem, self-perceived burden, coping resources, social support, sleep quality, fatigue, mental health service use and suicidal ideation (9, 16, 25–27, 30, 33, 36, 46).

**Figure 2:**
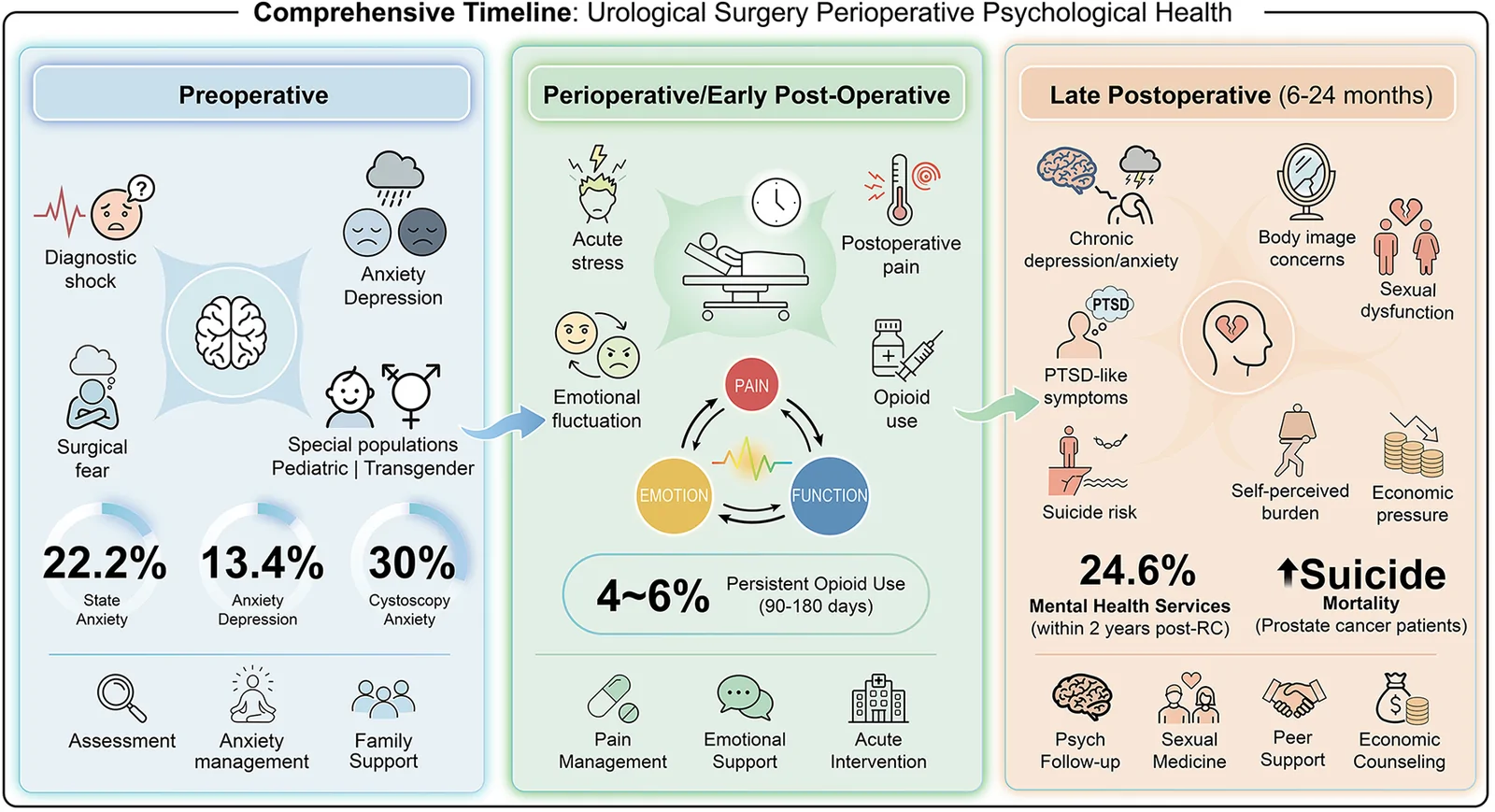
Integrated timeline of reported perioperative mental health concerns in urological and genitourinary surgery, illustrating concerns identified during the preoperative period, early postoperative recovery and longer-term follow-up, together with potential supportive considerations.

The diversity of measures indicates that perioperative mental health in urological and genitourinary surgery has been conceptualized in multiple ways, ranging from symptom screening to quality of life, coping resources, family support and service use. However, instruments, scoring approaches and assessment time points varied substantially across studies (7, 25, 30, 34–37, 39, 47). This heterogeneity limits direct comparison of prevalence estimates and makes pooled quantitative synthesis inappropriate. It also suggests that future care pathway research should define a smaller set of core psychological outcomes and measure them at repeated perioperative time points. This breadth of outcomes suggests that perioperative psychological assessment in urological and genitourinary surgery should extend beyond anxiety and depression alone and include function-related, psychosocial and context-sensitive domains relevant to public mental health-oriented care pathways. A detailed inventory of measurement tools is provided in Supplementary Table S2. The temporal pattern of reported perioperative psychological concerns across the surgical pathway is summarized in Table 1 and illustrated in Figure 2.

**Table 1:** Perioperative psychological issues in urology across different stages.

Perioperative stage	Main concerns	Reported evidence pattern
Preoperative phase (Diagnosis to Surgery)	•Anxiety •Depressive symptoms •Diagnostic and treatment uncertainty •Procedural fear •Information needs •and limited coping or support	•Anxiety, depression and traumatic stress symptoms were reported at diagnosis and before surgery, although estimates varied by disease, procedure and measurement instrument (7, 22, 30) •Patients undergoing diagnostic cystoscopy reported anxiety and depressive symptoms that were associated with greater procedure-related pain (47) •Patients undergoing major urogynecologic surgery had a median APAIS score of 24, with prominent recovery concerns and no measured demographic or clinical factors associated with higher anxiety (38) •Longer preoperative waiting and healthcare disruption were associated with greater anxiety, surgical fear or perceived stress (39, 45, 48) •Lower coping self-efficacy and perceived social support were associated with poorer mental health among individuals seeking vaginoplasty or vulvoplasty (16)
Perioperative and early postoperative (Intraoperative to 3 months postoperatively/until discharge)	•Pain-related distress •Sleep disturbance •Loss of control •Acute adjustment •Device-related discomfort	•Early psychological outcomes were commonly related to pain, sleep disruption and adjustment to altered urinary function or reconstruction (26, 40, 41, 46) •Stomas, urinary diversion and inadequate preparation were associated with emotional difficulty, loss of control and adaptation to new bodily routines (41, 46, 49) •Ureteral stent indwelling was associated with increased tension, helplessness and perceived psychological stress (50) •Anxiety and depressive symptoms were associated with greater procedure-related pain in diagnostic or locally anesthetized procedures (34, 47) •Perioperative opioid exposure was associated with new persistent or prolonged opioid use in a small subgroup of patients undergoing selected urological procedures (51, 52)
Mid-to-long-term postoperative (3–6 months to years)	•Persistent distress in a subgroup •Adjustment difficulties •Mental health service use •Depression and suicidality	•Quality of life and emotional wellbeing improved over time in several reconstructive, renal and rehabilitation cohorts, but recovery differed across procedures and patient subgroups (29, 43, 53, 54) •Persistent psychosocial distress remained in a subgroup of patients after radical cystectomy and urinary diversion at one- and 2-year follow-up (55, 56) •Poorer preoperative mental health and continuing urinary or sexual problems were associated with less favorable longer-term outcomes after prostate cancer treatment (42, 58, 59) •Social constraints and recurrent unwanted cancer-related thoughts were associated with poorer psychological wellbeing after prostate cancer surgery (57) •Mental health service use, depression, suicidal thinking and suicide risk were reported in selected urological cancer populations (36, 60–62)
Cross-stage specific issues (I) Body image, Sexuality and gender role	•Incontinence •Urinary diversion •Sexual dysfunction •Body-image change •Genital alteration •Masculinity and gender-related concerns	•Urinary diversion, stoma management and incontinence were associated with altered daily routines, body-image concerns, embarrassment and difficulties in social participation (40, 41, 46, 49, 55, 56) •Urinary leakage and sexual dysfunction remained important quality-of-life and adjustment concerns after prostate cancer treatment (58, 59) •Penile cancer studies identified reduced self-esteem, impaired sexual functioning, altered body image and challenges to masculine identity (27, 63, 64) •Gender-affirming surgery studies described coping, social-support, communication, healthcare-navigation and procedure-specific functional concerns (15, 16, 35, 65)
Cross-stage specific issues (II) SPB, finances and family	•Low social support •Social constraints •Self-perceived burden •Family-role disruption •Financial strain	•Greater social support was associated with lower anxiety or depression, whereas discouraging responses to discussion of illness were associated with poorer psychological wellbeing (22, 25, 30, 57) •Self-perceived burden was associated with poorer quality of life and socioeconomic or functional difficulties in one urological cancer cohort (66) •Parental psychological symptoms and family context were associated with postoperative quality of life and psychological wellbeing in pediatric urology (12) •Dependence, inadequate preparation and changes in daily or family roles contributed to emotional needs after cystectomy (49) •Living kidney donors generally reported favorable mental health-related quality of life, although complications, persistent pain, scar concerns or adverse recipient outcomes were associated with psychological difficulties in selected patients (37, 67)

COVID-19, coronavirus disease 2019; ED, erectile dysfunction; ECOG, Eastern Cooperative Oncology Group (performance status); GAS, gender-affirming surgery; HADS, Hospital Anxiety and Depression Scale; MHS, mental health services; PSS-4, 4-item Perceived Stress Scale; PTSD, post-traumatic stress disorder; QoL, quality of life; RC, radical cystectomy; RN, radical nephrectomy; RP, radical prostatectomy; SPB, self-perceived burden; STAI, State–Trait Anxiety Inventory; TGD, transgender and gender-diverse.

### 3.3 Temporal trajectory of perioperative psychological distress

#### 3.3.1 Preoperative phase

Preoperative psychological outcomes were examined predominantly in cross-sectional or single-time-point observational studies, with additional evidence from prospective surveys and qualitative interviews. Preoperative distress was most often reported in relation to new diagnosis, uncertainty about treatment outcomes and fear of the upcoming procedure. In patients undergoing surgery for urological cancer, psychological distress was already evident before treatment and was linked to cancer-related concerns, surgical uncertainty and anticipated postoperative consequences (7). Among patients treated for bladder cancer, depression and anxiety were associated with demographic, clinical and psychosocial factors, indicating that baseline psychological vulnerability and social context may shape distress before definitive treatment (22). In newly diagnosed bladder and kidney cancer, anxiety, depression and post-traumatic stress symptoms were also prominent, suggesting that psychological burden may begin at diagnosis rather than during postoperative recovery alone (30).

In patients undergoing diagnostic cystoscopy, approximately one-third reported anxiety and approximately one-quarter reported depressive symptoms; both anxiety and depressive symptoms were associated with greater procedure-related pain (47). In a mixed-methods study of 54 patients undergoing major urogynecologic surgery, the median preoperative APAIS score was 24. Recovery-related concerns predominated, and no measured patient characteristics were statistically associated with higher anxiety (38). Procedure-related organization also mattered: patients waiting longer on the surgical stretcher before urological and genitourinary surgery reported higher anxiety and surgical fear (39). During the COVID-19 pandemic, urological patients reported elevated perceived stress, illustrating how broader healthcare disruption can intensify perioperative psychological burden (48).

A qualitative interview study of 36 transgender adults undergoing gender-affirming surgery described prolonged preparation, the value of experienced and individualized surgical care, substantial self-advocacy demands, and barriers involving provider awareness, terminology and insurance coverage (15). Among transgender and gender-diverse individuals seeking vaginoplasty or vulvoplasty, lower coping self-efficacy and lower perceived social support were associated with poorer mental health (16). Overall, preoperative distress clustered around diagnosis, procedural fear, uncertainty, social support and baseline coping resources.

#### 3.3.2 Perioperative and early postoperative phase

In the perioperative and early postoperative phase, distress commonly shifted from anticipatory worry to more embodied recovery concerns, including pain, sleep disruption, acute adjustment and distress related to devices or altered urinary function. After radical cystectomy and urinary diversion, emotional recovery was shaped by adaptation to urinary reconstruction, perceived changes in body function and early postoperative symptom burden (46). One randomized controlled trial in living kidney donors reported lower perioperative anxiety and better sleep outcomes among participants receiving transcutaneous electrical acupoint stimulation (26).

Device-related experiences were a recurring source of distress. Patients undergoing cystectomy described emotional difficulty related to inadequate preparation, stoma-related change, loss of control and adaptation to new bodily routines (49). After pelvic exenteration involving cystectomy, urinary outcomes and quality of life varied by surgical approach, indicating that reconstruction type may influence functional and emotional recovery (40). Ureteral stent placement provided an important example of distress after a lower-trauma device-related intervention. In a prospective observational study, patients reported increased psychological stress during stent indwelling, including tension and helplessness, and perceived stress increased by approximately 66% during the stent-indwelling period compared with pre-procedural baseline (50).

Pain-related distress and opioid-related risk also emerged in early perioperative care. Diagnostic cystoscopy patients with anxiety or depression experienced greater procedure-related pain (47). One observational study of a mixed gender-affirming surgery cohort reported past-30-day cannabis use in 29 of 64 participants (45.3%), with concurrent associations with greater pain interference, anxiety and depressive symptoms; reasons for use and causality were not established (14). Evidence from urological procedural settings suggests that perioperative opioid exposure may contribute to new persistent opioid use in some previously opioid-naïve patients, including those undergoing male fertility procedures (51). A broader urological and genitourinary surgery analysis also identified opioid dependence and overdose as postoperative concerns in selected patients (52). Taken together, early postoperative distress was most often connected to pain, sleep, bodily control, device-related adaptation and behavioral health vulnerability.

#### 3.3.3 Intermediate and long-term postoperative phase

Longer-term evidence was derived mainly from prospective or retrospective cohort studies, repeated-measure rehabilitation studies and a small number of qualitative studies. At intermediate and longer-term follow-up, several studies suggested improvement in quality of life and emotional wellbeing for many patients, but recovery was heterogeneous across procedures and subgroups. In benign ureteral stricture reconstruction, minimally invasive reconstructive surgery was associated with favorable clinical and health-related quality-of-life outcomes (29). In renal cancer surgery, quality-of-life and perioperative outcomes were broadly maintained or improved after nephrectomy approaches in selected cohorts (53). In radical cystectomy and urinary diversion, rehabilitation data showed improvement in urinary symptoms, health-related quality of life and psychosocial distress during early recovery (54). However, 1- and 2-year follow-up studies after radical cystectomy and urinary diversion still identified persistent psychosocial distress in a subgroup of patients (55, 56).

Longer-term distress was often linked to functional sequelae and social context. Evidence after prostate cancer treatment included longitudinal patient-reported outcome studies and one qualitative study. Poorer preoperative mental health was associated with less favorable postoperative outcomes after robotic-assisted radical prostatectomy (42). A prospective study found that social constraints after prostate cancer surgery—defined as discouraging or negative responses from partners or others when patients attempted to discuss their cancer experience—were associated with poorer psychological wellbeing during follow-up (57). Patient-reported side effects 1 year after radical prostatectomy or radiotherapy included urinary and sexual problems that affected quality of life (58). One qualitative study described continuing concerns involving urinary leakage, sexual function, recovery uncertainty and return to usual daily life after radical prostatectomy (59). These findings indicate that long-term distress frequently coexists with continence problems, erectile dysfunction, social constraint and altered self-confidence.

A smaller but clinically important body of evidence pointed to more severe mental health outcomes and service-use needs in selected subgroups. Among patients undergoing curative-intent treatment for bladder cancer, approximately one-quarter received at least one mental health consultation within 2 years after treatment (60). In real-world data from urologic cancer patients, psychological suffering was associated with suicidal thinking (36). Large cohort studies in prostate cancer reported increased long-term risks of depression and suicide, particularly around vulnerable periods such as diagnosis and early treatment (61, 62). These findings do not indicate uniform long-term psychological deterioration across all urological patients; rather, they show that persistent distress and suicide risk are concentrated in identifiable subgroups.

### 3.4 Urology-relevant risk domains

#### 3.4.1 Urinary, sexual and body-image concerns

Evidence on cystectomy and urinary diversion included qualitative, observational, rehabilitation and longitudinal follow-up studies. Urinary diversion, stoma dependence, incontinence and erectile dysfunction were among the most distinctive psychological stressors in urological and genitourinary surgery. A qualitative study of patients undergoing cystectomy described emotional distress related to inadequate preparation, loss of control and adaptation to stoma care and new bodily routines (49). Observational studies of cystectomy, pelvic exenteration and urinary diversion reported concerns involving altered urinary function, body image, stoma-related embarrassment and social participation (40, 41, 46). Two longitudinal studies identified persistent psychosocial difficulties in a subgroup of patients 1–2 years after radical cystectomy and urinary diversion (55, 56). These studies indicate that urinary diversion is not only a physical or technical outcome, but also a source of emotional and social adjustment.

In prostate cancer treatment, urinary and sexual dysfunction were repeatedly associated with psychological outcomes. One year after radical prostatectomy or radiotherapy, patient-reported side effects included urinary and sexual problems that affected quality of life (58). Men after radical prostatectomy described ongoing needs related to urinary leakage, erectile dysfunction, uncertainty and social reintegration (59). Social support was associated with lower anxiety and depression through self-efficacy in patients with prostate cancer (25), while social constraints after surgery were prospectively associated with poorer psychological wellbeing over time (57). These findings suggest that functional recovery and psychological recovery are closely connected, particularly when urinary control and sexual function are central to identity, intimate relationships and confidence.

#### 3.4.2 Genital surgery and gender-related concerns

Cancer-related genital surgery and gender-affirming surgery involved different but overlapping concerns related to body image, sexual function and identity. In penile cancer, two cross-sectional studies reported impaired health-related quality of life, lower self-esteem and sexual-function concerns after surgery (27, 63), while one qualitative study described altered masculinity, intimacy and social participation after partial or total penectomy (64). These findings indicate that genital surgery may generate sensitive concerns that are unlikely to be disclosed unless clinicians ask about them directly and non-judgmentally.

Evidence on gender-affirming surgery was mainly observational and qualitative. In transfeminine individuals undergoing genital surgery, an individualized perioperative hormone-management approach was associated with stable behavioral health measures and did not show a significant increase in postoperative complications (35). Patient-reported outcome research on feminizing genital surgery showed generally favorable body image, quality-of-life, urinary, sexual and decision-related outcomes, while also identifying specific functional concerns relevant to recovery expectations (65). These findings identify communication, counseling and care-navigation domains that may be relevant to future perioperative support.

Qualitative evidence highlighted the value of individualized counseling and clinicians experienced in transgender health, alongside self-advocacy demands and barriers related to provider awareness, terminology and insurance coverage (15). Among individuals seeking vaginoplasty or vulvoplasty, lower coping self-efficacy and lower perceived social support were associated with poorer mental health (16). Pain and behavioral-health vulnerabilities may also warrant assessment in selected patients (14). Taken together, these studies suggest that perioperative mental health in gender-affirming surgery should be understood in relation to coping resources, pain, social support, sexual function and patient-clinician communication.

#### 3.4.3 Self-perceived burden, family role and financial stress

Self-perceived burden, family dependence and financial stress were important psychosocial domains across urological populations. One cross-sectional study of patients with urological cancer reported self-perceived burden in approximately 73% of participants and identified associations with poorer quality of life and socioeconomic or functional factors (66). Together, these findings suggest that perceived burden may be shaped by functional dependence and the availability of support during recovery (49, 66).

Family and partner relationships also shaped recovery. A qualitative cystectomy study described emotional needs related to dependence, inadequate preparation and changes in daily life (49). In prostate cancer, social support was associated with lower anxiety and depression through self-efficacy (25), whereas social constraints were prospectively associated with poorer psychological wellbeing during longer-term recovery (57). Pediatric urology provided another example of family-system influence: postoperative quality of life and psychological wellbeing were associated with parental psychological symptoms, previous surgeries and family context (12). Living-donor evidence came from prospective and follow-up cohorts that generally reported favorable mental health-related quality of life but identified psychological difficulties among selected donors with complications, persistent pain, scar concerns or adverse recipient outcomes (37, 67). Across these studies, family context could function either as a source of support or as a contributor to distress when dependence, financial pressure or communication difficulties were prominent.

#### 3.4.4 Cross-cutting risk factors

Risk factors for perioperative psychological distress were distributed across clinical, psychological, social and procedure-related domains. Clinical risk factors included malignancy, treatment uncertainty, disease burden, recurrence concerns and postoperative oncological or functional outcomes (22, 30, 32, 42, 44, 68). In newly diagnosed bladder and kidney cancer, psychological disorders were linked with psychosocial resources and the shock of cancer diagnosis (30). In prostate cancer after castration, anxiety and depression were associated with clinical and treatment-related factors, and risk prediction models were developed using patient characteristics and treatment variables (32). In radical cystectomy, poor preoperative mental health was associated with high-grade complications (69).

Baseline psychological vulnerability was another recurring risk domain. Poor preoperative mental health was prospectively associated with less favorable outcomes after robotic-assisted radical prostatectomy (42), while previous or ongoing mental health service use was relevant in bladder cancer treatment trajectories (60). Psychological suffering was associated with suicidal thinking in urologic cancer patients (36). Social risks included low social support, social constraint, financial strain and self-perceived burden (25, 57, 66). Procedure-related risks included urinary diversion, stoma dependence, incontinence, erectile dysfunction, genital surgery and device-related distress (40, 46, 49, 55, 56, 58, 59, 64). These risk domains often overlapped, meaning that distress was rarely explained by procedure type alone (Table 2).

**Table 2:** Major risk factors and mechanisms of perioperative psychological distress in urology.

Risk domain	Representative factors	Synthesis of included evidence
Baseline psychological and demographic context	•Previous anxiety or depression •Poor self-rated mental health •Prior mental health service use •Age •Sex •Marital status •Previous treatment experience	•Poor baseline mental health was associated with less favorable postoperative outcomes or higher-grade complications in selected prostatectomy and cystectomy cohorts (42, 69) •Previous or continuing mental health service use was associated with persistent psychological needs after bladder cancer treatment (36, 60) •Associations involving age, sex, marital status, education and previous surgery varied across populations and were not consistently reproduced (7, 22, 30, 32, 47, 50) •In major urogynecologic surgery, substantial anxiety and recovery-related concerns were reported, but no measured demographic or clinical variable was statistically associated with higher anxiety (38)
Disease, prognosis and treatment uncertainty	•New cancer diagnosis •Disease burden •Prognosis •Recurrence concerns •Adverse oncological findings	•New diagnosis, treatment uncertainty and perceived threat were associated with anxiety, depression, traumatic stress symptoms or maladaptive adjustment (7, 9, 22, 30) •Concerns regarding treatment effectiveness, recurrence and postoperative oncological outcomes were associated with greater distress in selected studies (32, 44, 68) •Anticipated urinary, sexual or functional consequences contributed to distress and information needs before definitive treatment (7, 38, 49)
Functional, anatomical and device-related change	•Urinary diversion •Stomas •Incontinence •Sexual dysfunction •Genital alteration •Catheters •Ureteral stents	•Urinary diversion, stoma care and altered urinary function were associated with emotional adjustment, body-image concerns and reduced social participation (40, 41, 46, 49, 55, 56) •Ureteral stent indwelling was associated with increased tension, helplessness and perceived psychological stress (50) •Urinary leakage and sexual dysfunction were continuing quality-of-life and adjustment concerns after prostate cancer treatment (58, 59) •Penile cancer studies described lower self-esteem, impaired sexual functioning, altered body image and challenges to masculine identity (27, 63, 64)
Pain and behavioral-health vulnerability	•Procedure-related pain •Chronic pain •Psychiatric comorbidity •Substance-use history •Opioid exposure	•Psychological distress and poorer baseline mental health were associated with greater pain or less favorable recovery outcomes in selected studies (42, 47, 69) •One observational study of gender-affirming surgery reported concurrent associations among recent cannabis use, pain interference, anxiety and depressive symptoms, without establishing causality (14) •Perioperative opioid exposure and psychiatric, pain or substance-use vulnerability were associated with persistent opioid use or opioid-related complications in selected urological populations (51, 52)
Social, family and socioeconomic context	•Low social support •Social constraints •Family strain •Financial stress •Self-perceived burden •Parental psychological symptoms	•Lower perceived social support was associated with poorer mental health, whereas greater support was associated with lower anxiety or depression in selected populations (16, 22, 25, 30) •Social constraints and recurrent unwanted cancer-related thoughts were prospectively associated with poorer psychological wellbeing after prostate cancer surgery (57) •Self-perceived burden was associated with poorer quality of life and socioeconomic or functional difficulties in one urological cancer cohort (66) •Parental psychological symptoms and family context were associated with postoperative quality of life and psychological wellbeing in pediatric urology (12) •Living-donor studies identified psychological difficulties in selected participants with complications, persistent pain, scar concerns or adverse recipient outcomes (37, 67)
Communication, preparation and information needs	•Inadequate preparation •Unmet expectations •Limited participation in decision-making •Self-advocacy •Healthcare-navigation barriers	•Patients reported unmet information needs concerning the procedure, recovery, urinary or sexual function and postoperative self-care (38, 40, 41, 46, 49, 59) •Inadequate preparation and limited involvement in treatment or recovery planning were associated with emotional difficulty and continuing support needs (49, 59, 73) •Transgender adults emphasized individualized communication, clinicians experienced in transgender health and reduced reliance on patient self-advocacy when navigating perioperative services (15)

The domains are not mutually exclusive, and individual studies could contribute to more than one domain. Unless otherwise stated, the summarized findings represent descriptive or associative evidence rather than established causal relationships.

### 3.5 Supportive interventions and care pathway implications

#### 3.5.1 Preoperative preparation and prehabilitation

Prehabilitation studies suggested that preoperative preparation may influence both physical and psychological readiness. In patients scheduled for radical cystectomy for bladder cancer, presurgical exercise interventions were feasible and may support structured physical preparation, with some evidence of improved general and mental health-related quality-of-life outcomes (70, 71). A randomized trial of multimodal prehabilitation combining exercise, nutritional counseling and relaxation reported favorable postoperative functional-capacity outcomes (72). Although mental health was not always the primary outcome, these studies suggest that physical preparation, expectation setting and perceived control may be connected in the preoperative phase.

Structured education and modified perioperative management models were also reported. A retrospective controlled study after radical prostatectomy reported more favorable mental-state, quality-of-life and self-care outcomes among patients receiving a modified perioperative management model (31). A cross-sectional patient-experience survey found that structured education and written materials were highly valued after urogynecologic surgery (73). A separate mixed-methods observational study found that patients perceived preoperative counseling and confidence in the surgical team as helpful for managing anxiety, although no intervention was formally tested (38). These studies suggest that preoperative preparation may be most useful when it combines information, expectation management, functional preparation and self-management support.

#### 3.5.2 Pathway-integrated supportive care and multidisciplinary follow-up

Evidence for nursing-led and pathway-based care came primarily from single-center retrospective, non-randomized or controlled studies of multicomponent interventions. Several studies evaluated nursing-led or multidisciplinary perioperative models. A Roy Adaptation Model-based perioperative care intervention in older adults with benign prostatic hyperplasia was associated with improved psychological wellbeing, pain and quality of life (28). In kidney transplant recipients, an innovative multidisciplinary perioperative nursing model combining team-based support, Rosenthal effect-based psychological care, Enhanced Recovery After Surgery protocols and 3-month digital follow-up was associated with lower anxiety, better mental health-related quality of life and improved recovery-related outcomes than standard care (33). Watson's Caring Model after radical prostatectomy was associated with improved self-esteem, depression and resilience (74).

Continuing-care interventions extended support beyond hospital discharge. Mobile internet management after radical prostatectomy was associated with improved continuing care and may help maintain education, symptom monitoring and self-management support after discharge (75). These studies indicate that psychological support can be incorporated into routine perioperative care, multidisciplinary coordination and post-discharge follow-up, with nursing teams contributing to education, symptom monitoring and continuity of care. However, the heterogeneity and limited methodological strength of these studies mean that their findings should be interpreted cautiously.

#### 3.5.3 Digital, VR and non-pharmacological strategies

Digital and non-pharmacological strategies were evaluated in a small number of controlled or feasibility studies. A multicenter feasibility randomized controlled trial evaluated a digital stress-reduction program in mixed surgical populations, including patients undergoing urological cancer surgery, and reported potentially favorable changes in stress, anxiety, negative affect and postoperative pain (24). One procedure-specific study reported that virtual reality during functional urological procedures under local anesthesia was feasible and acceptable, with potentially favorable patient-experience, anxiety or pain outcomes (34). One small single-center randomized controlled trial in living kidney donors reported lower perioperative anxiety, better sleep and favorable short-term recovery outcomes among participants receiving TEAS compared with sham treatment (26). Overall, these studies were small, heterogeneous and procedure-specific; therefore, they indicate feasibility or potential benefit rather than established effectiveness or generalizability across urological surgery.

#### 3.5.4 Risk-stratified follow-up and referral

Several observational and follow-up studies identified characteristics that may be relevant to future risk-stratified follow-up, including poor baseline mental health, persistent postoperative distress, previous mental health service use, severe pain and functional difficulties. Psychological distress improved during recovery in some cohorts but persisted in a subgroup of patients, particularly after cystectomy and urinary diversion (44, 54–56). Poor baseline mental health, previous mental health service use and persistent distress were identified as potential markers of continuing support needs (23, 36, 42, 60, 69). These findings may inform the future development of assessment and referral pathways but do not constitute a validated risk-stratification model.

Parallel evidence identified psychiatric comorbidity, chronic pain, substance use and other vulnerabilities as potential considerations when developing pain follow-up and opioid-stewardship approaches (51, 52). Continuing psychosexual, body-image and functional support needs were reported among patients undergoing urinary diversion, prostate or penile cancer surgery and gender-affirming surgery (15, 16, 27, 49, 59, 63–65). Candidate components for future pathway evaluation include repeated assessment, tailored education, referral coordination and longer-term supportive follow-up (Table 3).

**Table 3:** Overview of perioperative psychological intervention strategies and evidence.

Supportive approach	Typical components	Synthesis of included evidence
Preoperative education and prehabilitation	•Exercise •Nutritional and relaxation components •Procedure and recovery information •Expectation management	•Preoperative exercise and multimodal prehabilitation were feasible and were associated with favorable functional or selected quality-of-life outcomes after radical cystectomy (70–72) •Structured education, written information and preparation for postoperative self-care were valued by patients undergoing prostate or urogynecologic surgery (38, 59, 73) •Patients undergoing major urogynecologic surgery identified preoperative discussion, teaching and confidence in the surgical team as helpful, although no educational intervention was formally tested (38) •Psychological outcomes were often secondary, and the contribution of individual exercise, nutritional, educational or relaxation components could not be determined
Nursing-led and pathway-based care	•Structured assessment •Emotional support •Coping guidance •Family involvement •Enhanced recovery •Self-care training and post-discharge follow-up	•Roy Adaptation Model-based care and modified perioperative management were associated with improvements in anxiety, depression, pain, quality of life or self-care in selected studies (28, 31) •A multidisciplinary perioperative nursing model in kidney transplantation was associated with favorable anxiety, sleep, mental health-related quality-of-life, adherence and recovery outcomes (33) •Watson's Caring Model and mobile continuing-care programs after radical prostatectomy were associated with improved psychological or continuity-of-care outcomes (74, 75) •Most interventions were single-center, retrospective or multicomponent, limiting attribution of effects to individual elements
Digital, virtual reality and non-pharmacological strategies	•Digital stress-reduction tools •Virtual reality relaxation or distraction •Transcutaneous electrical acupoint stimulation	•A feasibility randomized trial of a digital stress-reduction intervention reported potentially favorable short-term changes in stress, anxiety, negative affect and postoperative pain (24) •Virtual reality during procedures under local anesthesia was feasible and acceptable and was associated with potentially favorable anxiety, pain or patient-experience outcomes (34) •One randomized trial in living kidney donors reported lower perioperative anxiety, improved sleep and favorable short-term recovery outcomes following transcutaneous electrical acupoint stimulation (26) •Evidence was based on a small number of procedure-specific studies and does not establish effectiveness across urological surgery
Repeated assessment, risk stratification and longitudinal follow-up	•Screening before surgery •Reassessment during recovery •Monitoring of pain, support and service use •Referral triggers	•Poor baseline mental health, depressive symptom severity, pain and previous mental health service use were identified as potential markers of greater postoperative need (32, 36, 42, 60, 69) •Rehabilitation and longitudinal studies showed improvement for many patients but persistent distress in selected subgroups after cystectomy, urinary diversion and prostate cancer treatment (44, 54–56) •The absence of clear predictors in the urogynecologic cohort suggests that routine patient characteristics alone may not be sufficient to identify all patients with substantial preoperative anxiety (38) •No validated cross-procedure prediction model, screening threshold or referral algorithm was established
Family, communication and psychosexual support	•Partner or family involvement •Supportive communication •Opportunities to discuss illness •Sexual-health, body-image and gender-affirming support	•Social support was associated with better psychological outcomes, whereas social constraints and inadequate preparation were associated with poorer adjustment (12, 25, 49, 57). •Continuing sexual, body-image and identity-related support needs were reported after prostate, penile, urinary-diversion and gender-affirming surgery (15, 16, 27, 59, 63–65) •Patients valued individualized information, experienced clinicians and respectful healthcare interactions, particularly in gender-affirming and genital surgery settings (15, 38) •Direct comparative trials of family, communication or psychosexual interventions were scarce
Pain management and opioid stewardship	•Pain assessment and communication •Multimodal or opioid-sparing analgesia •Monitoring of psychiatric, pain or substance-use vulnerability	•Anxiety and depressive symptoms were associated with greater procedural or postoperative pain in selected observational studies (14, 47) •Persistent opioid use occurred in a small subgroup of patients following perioperative opioid exposure in selected urological procedures (51, 52) •The evidence identifies pain communication, monitoring and opioid-stewardship as considerations but does not establish the effectiveness of a specific psychological or opioid-prevention intervention

CBT, cognitive behavioral therapy; ERAS, Enhanced Recovery After Surgery; GAS, gender-affirming surgery; HADS, Hospital Anxiety and Depression Scale; PHQ-9, Patient Health Questionnaire-9; RAM, Roy Adaptation Model; SF-12/36, 12-/36-Item Short Form Health Survey; TEAS, transcutaneous electrical acupoint stimulation; VR, virtual reality.

## 4 Discussion

### 4.1 Principal findings

This scoping review shows that perioperative mental health in urological and genitourinary surgery should not be understood as a single emotional reaction occurring around the time of surgery. Rather, psychological distress appears to be staged, context-sensitive and closely related to the distinctive functional and bodily consequences of urological procedures. Across the included literature, distress commonly emerged before surgery in relation to uncertainty, fear and treatment decision-making; during early recovery in relation to pain, sleep disruption and device-related discomfort; and during longer-term recovery in relation to urinary function, sexual function, body image, family roles and social participation (7, 44, 49, 55, 57).

A central finding is that urological and genitourinary surgery creates psychosocial stressors that are not fully captured by generic surgical anxiety or depression frameworks. Urinary diversion, stomas, catheters and altered urinary function may affect privacy, independence and daily self-management (40, 41, 46, 49, 55). Erectile dysfunction, genital surgery and altered genital appearance may affect intimacy, masculinity, self-esteem and willingness to seek help (27, 59, 64). These concerns are clinically important because they may be concealed by patients, normalized as unavoidable surgical consequences, or missed unless clinicians ask proactively.

The review also found that intervention evidence is less developed than descriptive evidence. Prehabilitation, structured education, digital or virtual reality strategies, TEAS, nursing models and rehabilitation-based follow-up suggest potentially useful approaches, but the current evidence remains heterogeneous and is often limited by small samples, observational designs or single-center settings (24, 26, 28, 33, 36, 70). The literature therefore identifies candidate components for pathway-oriented supportive care but does not establish a definitive intervention model.

### 4.2 Interpretation through a staged care-pathway perspective

**Figure 3:**
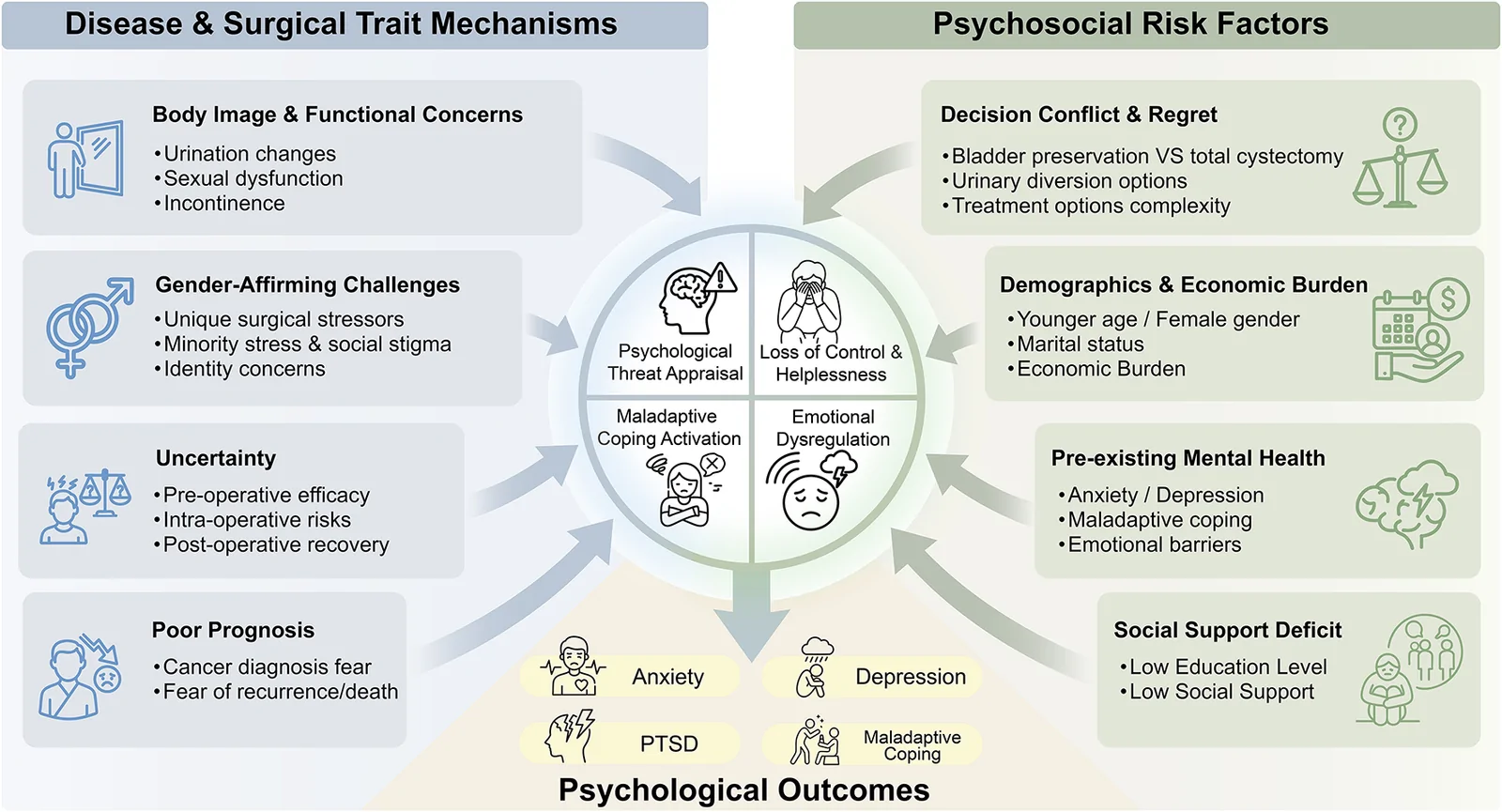
Provisional conceptual framework of potential contributors to perioperative psychological distress in urological and genitourinary surgery. The framework is an interpretive synthesis rather than a validated predictive or causal model; arrows indicate proposed relationships for future evaluation.

We developed a provisional staged framework from the descriptive synthesis to summarize recurring patterns and identify potential assessment points (Figure 3). It is interpretive rather than validated and is intended for hypothesis generation and future evaluation. In line with transactional stress theory, distress may be understood in relation to perceived threat, perceived control and available coping resources (76, 77). Patients must anticipate possible changes in survival, urinary function, sexual function, body image and social identity before they have direct experience of recovery. At this stage, structured information, expectation management and shared decision-making may reduce uncertainty and improve perceived control (30, 42, 47, 48).

After surgery, distress becomes more closely connected to embodied recovery experiences. Pain, fatigue, sleep disruption, catheters, stomas or ureteral stents can make surgical consequences immediate and concrete. These experiences may reinforce one another: pain may worsen anxiety, anxiety may disrupt sleep, and poor sleep may further reduce coping capacity. These findings provide a rationale for evaluating integrated approaches combining psychological support, symptom management, device education and early rehabilitation (42, 49, 51).

In the medium- and long-term phase, psychological outcomes were frequently reported in relation to adaptation to functional, sexual and social consequences. Persistent incontinence, erectile dysfunction, urinary diversion or altered genital appearance may be experienced not only as physical problems but also as threats to intimacy, attractiveness, independence or social roles (55, 57, 59). These findings provide a rationale for evaluating mental health support beyond hospital discharge within survivorship, rehabilitation and functional follow-up.

### 4.3 Clinical and care pathway implications

The findings of this review may inform the future development of more proactive, pathway-based approaches to perioperative psychological support. Potential assessment points include the preoperative period, early recovery, discharge or rehabilitation, and longer-term follow-up, because the content of distress may change over time (12, 14, 30, 42, 47, 50). In one urogynecologic cohort, routine demographic and clinical characteristics did not identify patients with higher anxiety, suggesting that these variables alone may be insufficient for targeted case identification (38). Psychological assessment could therefore be evaluated alongside perioperative risk stratification, discharge planning, rehabilitation and survivorship care. Recent cross-disciplinary surgical literature has similarly emphasized the potential value of embedding psychological care within surgical treatment plans, incorporating preoperative psychological screening, targeted intervention and longitudinal follow-up (78). These proposals are conceptually promising and warrant further urology-specific implementation and effectiveness research.

In early recovery, future pathway studies could evaluate coordinated psychological assessment alongside pain, sleep, symptom and device management. Candidate support domains at discharge and follow-up include self-care confidence, continence or stoma management, family involvement, sexual adjustment, body image and continuity of care (49–51, 54, 66, 73). Candidate pathway components include defined assessment points, referral triggers, patient education, family-inclusive support and communication across inpatient, outpatient, rehabilitation and community settings.

Evidence from non-urological chronic-disease populations provides proof-of-concept for more structured psychological interventions (79–82). Group cognitive-behavioral therapy has been associated with reductions in anxiety and depressive symptoms and with improvements in quality of life among people with Parkinson's disease (80, 81), while virtual reality-assisted group CBT has also shown potential to improve emotional wellbeing and psychological symptoms (79, 82). These findings do not establish effectiveness in perioperative urological care; rather, they identify candidate intervention components—including CBT-based coping skills, group delivery, immersive relaxation or rehearsal, and digitally supported follow-up—that warrant adaptation and testing within urological surgical pathways (79–82).

Although this review should not be framed solely as a nursing review, many of the identified needs are nursing-sensitive and therefore have direct implications for public mental health service delivery within perioperative care. Nurses are often closely involved in education, symptom monitoring, device care, continence support, discharge preparation and follow-up communication. This places them in a strong position to detect distress, normalize sensitive concerns and initiate referral. However, these responsibilities should be embedded within multidisciplinary and system-level pathways rather than relying on individual clinician awareness alone. Psycho-oncology, psychology or psychiatry may be needed for severe distress, suicidal ideation or previous mental health service use (36, 60, 61). Pain services may be needed for persistent pain or opioid vulnerability (14, 51, 52, 56). Sexual medicine or psychosexual counseling may be needed for sexual and body-image concerns (27, 59, 63, 64). Social work, family support or community-based services may be needed for high self-perceived burden, financial strain or poor support (12, 25, 66). From a public health perspective, predefined referral routes across surgical, rehabilitation and follow-up services may facilitate the early identification of psychological distress and access to appropriate, acceptable and sustainable support.

### 4.4 Future research directions

Future research should move beyond describing distress toward developing and testing pathway-based models of care. First, studies should use more consistent psychological and patient-centered outcome measures. Current evidence is difficult to compare because studies vary in instruments, cut-offs, timing and outcome domains. A core outcome set for perioperative mental health in urological and genitourinary surgery would improve comparability across procedures and allow stronger synthesis in future reviews.

Second, more longitudinal studies are needed. Perioperative distress should be assessed at predefined time points, including preoperative baseline, early postoperative recovery, discharge or rehabilitation, and longer-term follow-up. Such studies should examine not only anxiety and depression, but also uncertainty, perceived control, pain-related distress, sleep, self-efficacy, body image, sexual adjustment, social support, self-perceived burden and discharge readiness.

Third, intervention research should become more pragmatic and implementation-oriented. Future trials should test integrated care pathways that combine screening, education, family-inclusive support, symptom monitoring, digital follow-up, referral triggers and multidisciplinary rehabilitation. These studies should evaluate not only symptom improvement but also feasibility, acceptability, sustainability, resource requirements and cost-effectiveness across different healthcare settings (24, 28, 33, 70).

Finally, future research should include underrepresented populations and contexts. Evidence remains relatively limited for pediatric urology, benign reconstructive procedures, living kidney donation, kidney transplantation, gender-affirming surgery and low-resource settings (25, 35, 40). Intervention development should also be culturally responsive. A recent Iranian framework highlights the influence of individual, family, institutional and cultural factors on mental health (83). In urological surgery, masculinity, sexuality, stigma and family roles may similarly influence help-seeking and engagement with supportive care (25, 57, 64, 66). Perioperative pathways should therefore be locally adapted and co-designed with relevant stakeholders.

### 4.5 Strengths and limitations

This review has several strengths. It synthesizes evidence across a broad urological spectrum and organizes findings according to the perioperative timeline. It also highlights specialty-specific psychological concerns that may be less visible in general surgical mental health literature, including urinary, sexual, body-image, device-related and family-role distress. By combining evidence mapping with a staged conceptual interpretation, the review provides a framework for linking psychological needs to screening, supportive care and referral.

Several limitations should be acknowledged. The included studies were heterogeneous in design, population, procedure type, assessment timing and psychological measures, which limited direct comparison and precluded quantitative synthesis. The proposed patterns should not be assumed to apply uniformly across all urological and genitourinary populations. The evidence base was weighted toward adult oncological cohorts, particularly bladder and prostate cancer, whereas pediatric, benign reconstructive, transplant, living-donor, urogynecologic and gender-affirming populations were less well-represented. Differences in healthcare systems, cultural contexts and access to mental health or follow-up services may further limit transferability across settings. Although an *a priori* protocol was developed before study screening, it was not prospectively registered or publicly deposited, limiting external verification of adherence to the original protocol. Most of the evidence was observational, and intervention studies were relatively few and heterogeneous; therefore, causal inferences and conclusions about intervention effectiveness remain limited. Evidence concerning cannabis use was derived from only one small observational study of a mixed gender-affirming surgery cohort, precluding procedure-specific or causal conclusions. The review was restricted to English-language publications, which may have resulted in the omission of relevant nursing, allied health and psychological literature. In keeping with scoping review methodology, no formal risk-of-bias or certainty-of-evidence assessment was undertaken; consequently, the findings should not be interpreted as evidence-graded clinical recommendations.

## 5 Conclusions

Perioperative mental health concerns were reported across a range of urological and genitourinary procedures, although their frequency, severity and timing varied across populations and settings. For descriptive purposes, the available evidence could be organized into a provisional staged pattern encompassing preoperative uncertainty and fear, early postoperative symptom- and device-related distress and longer-term functional and psychosocial adjustment. These findings may inform the future development and prospective evaluation of perioperative mental health pathways incorporating repeated assessment, risk stratification, structured education, family-inclusive support and referral mechanisms within routine surgical, rehabilitation and follow-up services. Nurses contribute substantially to assessment, education, symptom monitoring and care coordination, and these roles could be incorporated within broader multidisciplinary pathways involving urology teams, mental health professionals, pain services, sexual medicine, rehabilitation, social work and community-based support. The applicability of these findings is greatest for the adult oncological populations represented in the included literature and remains uncertain for underrepresented procedures, age groups and healthcare settings. These components require prospective validation before routine implementation.

## Data Availability

The original contributions presented in the study are included in the article/Supplementary material, further inquiries can be directed to the corresponding author.
