# Supplementary material for: Perioperative mental health needs in urological and genitourinary surgery: a scoping review
Source: Front Public Health. 2026 Aug 13;14:1904213. doi: 10.3389/fpubh.2026.1904213 (PMC13518201; doi:10.3389/fpubh.2026.1904213)
Supplement: Supplementary file 1 [file Table_1.docx]

Supplementary Appendix S1. Complete Search Strategies

The final systematic search was conducted in six bibliographic or full-text databases: PubMed, Embase, ScienceDirect, Web of Science Core Collection, APA PsycINFO and CINAHL Complete. Searches were limited to English-language publications published from January 2000 to the final search in May 2026.

The initial search formulation combined perioperative terms, mental health terms and a mandatory urological or genitourinary concept block. During search testing and supplementary citation checking, potentially eligible studies were identified whose urological relevance was not explicitly represented in their titles, abstracts or database indexing. The search strategy was therefore amended to improve sensitivity. In the final search, the mandatory urological or genitourinary block was removed, and urological relevance was assessed during title, abstract and full-text screening according to the predefined eligibility criteria. The review question and eligibility criteria were not changed.

- PubMed

**Platform:** PubMed, National Library of Medicine

**Date last searched:** May 2026

**Search field:** Title/Abstract

**Limits:** English language; publication date from January 2000 to the final search in May 2026

**Search strategy:**

(("Perioperative Period"[Title/Abstract] OR "Perioperative"[Title/Abstract] OR "Period, Perioperative"[Title/Abstract] OR "Periods, Perioperative"[Title/Abstract] OR "Perioperative Periods"[Title/Abstract]) AND ("Mental Health"[Title/Abstract] OR "Health, Mental"[Title/Abstract] OR "Mental Hygiene"[Title/Abstract] OR "Hygiene, Mental"[Title/Abstract]))

**Records identified:** n = 454

- Embase

**Platform:** Embase.com, Elsevier

**Date last searched:** May 2026

**Search field:** Title/Abstract

**Limits:** English language; publication year 2000-2026; search completed in May 2026

**Search strategy:**

('perioperative period':ti,ab OR 'perioperative':ti,ab OR 'period, perioperative':ti,ab OR 'periods, perioperative':ti,ab OR 'perioperative periods':ti,ab) AND ('mental health':ti,ab OR 'health, mental':ti,ab OR 'mental hygiene':ti,ab OR 'hygiene, mental':ti,ab)

**Records identified:** n = 673

- ScienceDirect

**Platform:** ScienceDirect, Elsevier

**Date last searched:** May 2026

**Search field:** ScienceDirect search interface

**Limits:** English language; publication date from January 2000 to the final search in May 2026

**Search strategy:**

("Perioperative Period" OR "Perioperative" OR "Period, Perioperative" OR "Periods, Perioperative" OR "Perioperative Periods") AND ("Mental Health" OR "Health, Mental" OR "Mental Hygiene" OR "Hygiene, Mental")

**Records identified:** n = 182

- Web of Science Core Collection

**Platform:** Web of Science, Clarivate

**Date last searched:** May 2026

**Search field:** Topic (title, abstract, author keywords and Keywords Plus)

**Limits:** English language; publication year 2000-2026; search completed in May 2026

**Search strategy:**

TS=("Perioperative Period" OR "Perioperative" OR "Period, Perioperative" OR "Periods, Perioperative" OR "Perioperative Periods") AND TS=("Mental Health" OR "Health, Mental" OR "Mental Hygiene" OR "Hygiene, Mental")

**Records identified:** n = 560

- APA PsycINFO

**Platform:** Ovid

**Date last searched:** May 2026

**Search field:** Title/Abstract

**Limits:** English language; publication year 2000-2026; search completed in May 2026

**Search strategy:**

1. ("perioperative period" or perioperative or "period, perioperative" or "periods, perioperative" or "perioperative periods").ti,ab.
2. ("mental health" or "health, mental" or "mental hygiene" or "hygiene, mental").ti,ab.
3. 1 and 2
4. limit 3 to English language
5. limit 4 to yr="2000 - 2026"

**Records identified:** n = 43

- CINAHL Complete

**Platform:** EBSCOhost

**Date last searched:** May 2026

**Search field:** Title (TI) and Abstract (AB)

**Limits:** English language; publication date from January 2000 to the final search in May 2026

**Search strategy:**

S1 TI ("perioperative period" OR perioperative OR "period, perioperative" OR "periods, perioperative" OR "perioperative periods") OR AB ("perioperative period" OR perioperative OR "period, perioperative" OR "periods, perioperative" OR "perioperative periods")
S2 TI ("mental health" OR "health, mental" OR "mental hygiene" OR "hygiene, mental") OR AB ("mental health" OR "health, mental" OR "mental hygiene" OR "hygiene, mental")
S3 S1 AND S2
Limiters: English Language; Published Date: January 2000-May 2026

**Records identified:** n = 117

Search yield and record management

A total of 2,029 records were identified before deduplication: PubMed (n = 454), ScienceDirect (n = 182), Embase (n = 673), Web of Science Core Collection (n = 560), APA PsycINFO (n = 43) and CINAHL Complete (n = 117). Before screening, 1,064 records were removed, comprising 1,059 duplicate records, one record outside the predefined publication-date range and four non-English records. A total of 965 records were screened by title and abstract.
